# Analysis of College Course Scheduling Problem Based on Ant Colony Algorithm

**DOI:** 10.1155/2022/7918323

**Published:** 2022-08-25

**Authors:** Ruqun Ge, Jingyi Chen

**Affiliations:** HoHai University, Nanjing 211100, China

## Abstract

Ant colony algorithm is a new evolutionary algorithm, which is gradually applied due to its easy robustness with other methods and excellent distributed computing mechanism. Currently, the application field of ant colony algorithm has been infiltrated by a single TSP problem. Ant colony algorithm moves the algorithm toward the optimal solution through the combination of positive feedback and negative feedback. This paper briefly analyzes the basic characteristics of the basic idea and principle of the ant colony algorithm to apply it to the ant colony problem. Abstract the course scheduling problem, transform the course arrangement problem into the maximum matching problem of solving the bipartite diagram, and discharge the high-quality curriculum that basically meet the needs.

## 1. Introduction

### 1.1. Background

Class scheduling problem is a typical multi-objective combination optimization problem involving a variety of factors [1–4]. We should not only consider the learning effect of students, but also take care of the reasonable work arrangement of teachers and make full use of teaching equipment [5]. Scheduling the class schedule is a very important and very complicated task for the university academic affairs office. Due to the large amount of courses in each school, manual scheduling will inevitably appear all kinds of conflicts, and its shortcomings are more and more prominent. With the development of computer science and technology, the use of computer scheduling will solve the disadvantages of manual scheduling [1, 6, 7]. It is the computer method of simulating the human brain with the computer, discussing the thinking law and the choice method of arranging the class schedule. Automatic class scheduling has the advantages of short class scheduling time, high quality, and manpower saving [8]. At present, the methods to solve the scheduling problem include genetic algorithm, expert system, binary graph and coloring theory, simulated annealing algorithm, backtracking method, and so on. However, from the actual use situation, the developed class scheduling software is not generally practical. On the one hand, the class scheduling system is a very complex system, and it is very difficult to solve all the constraints and aspects. On the other hand, because every kind of school or even every school has its own particularity, it is difficult to use the class scheduling software widely. Therefore, the current practice is still to develop a different class scheduling software for different types of schools and then take a separate manual adjustment method for inappropriate scheduling methods.

### 1.2. Analysis of Class Scheduling Problems

#### 1.2.1. Five Elements of the Lesson Scheduling Problem

The scheduling process involves the five elements of curriculum, teacher, class, classroom, and time, which are defined as the following set [9, 10]:

1. Curriculum collection: *O* = {*O*_1_, *O*_2_, *O*_3_,…, *O*_*L*_}, where *L* is the number of courses offered.
2. Teaching teacher collection: *T* = {*T*_1_, *T*_2_, *T*_3_,…, *T*_*K*_}, where *K* is the number of teachers taught throughout the school.
3. Class collection: *L* = {*L*_1_, *L*_2_, *L*_3_,…, *L*_*N*_}, where *N* is the number of classes in the whole school.
4. Classroom collection: *R* = {*R*_1_, *R*_2_, *R*_3_,…, *R*_*P*_}, where *P* is the total number of different types of classrooms.
5. Time collection: *I* = {*I*_1_, *I*_2_, *I*_3_,…, *I*_*Q*_}, where *Q* represents the number of time slices.

#### 1.2.2. Constraints for Class Scheduling Problems

The hard and soft constraints are as follows [11]:

Hard constraints

1. The classroom can only have one course at the same time.
2. The class can only have one course at the same time.
3. The teacher can only arrange one course at the same time.
4. The teacher can only teach in one class at the same time, except for joint classes.
5. The teaching classroom capacity must be greater than or equal to the number of the class.
6. The classroom type should match the type of course requirements for the classroom.
7. The total number of school classrooms must be greater than or equal to the total number of classes taught at the same time.

Soft constraint

1. Classes in the same class should be evenly distributed over a week [12].
2. Mathematics courses or professional courses are arranged in the morning, and elective courses are installed in the evening or on weekends.
3. In continuous classes or teaching, the distance between classrooms should not be too far.
4. Physical education class is generally arranged in the afternoon, and no other classes are arranged after the physical education class.
5. The same class is arranged in the same class, at least one day apart, but not more than two days.
6. The same class is arranged in the same class, at least one day apart, but not more than two days.
7. Teachers' teaching tasks are evenly distributed within a week.
8. The same teacher teaches the same course and different classes, and on the same day, each class arranges the teaching content to ensure the continuity of teaching.

## 2. An Overview of the Colony Algorithm

### 2.1. Basic Idea of the Algorithm

#### 2.1.1. Basic Thought

When an ant goes to m city for food, it randomly selects one city as the starting point, and then goes to all other cities one by one. The ant volatilizes pheromones in the process of foraging, and finally returns to the starting city. The ant can only arrive in each city once [13], What order does the ant enter the city to make the foraging journey shortest?

#### 2.1.2. Building a Mathematical Model of the Ant Colony Algorithm

Assuming the scale of the solving problem, where the foraging city number uses *m* instead, in the colony, the number of ants was n. Then get the city set *V*={*v*_1_, *v*_2_,…, *v*_*m*_}. Then, the resulting set of paths between the cities is *E* = {(*r*,*s*)|*r*,*s* ∈ *V*}. At time t, if *c*_*i*_(*t*) (*i* = 1,…, *m*) is the number of ants located in the city *i*, the total number of ants is represented by n, and then *n* is calculated as below:

(1) $$\begin{array}{c} n=\sum_{i=1}^{m}c_{i}\left(t\right). \end{array}$$

The concepts of taboo list, pheromone, and visibility emerged in the ant colony algorithm. Taboo list is a data structure that records the city that ants pass through in order to avoid ants entering the same city many times. Tubek is the taboo table of ant *k*, ant *k* passes through city *i* and adds city *i* to the taboo table Tubek. Tubek(*q*) is the *q* element in the taboo table of the ant *k*, indicating the *q* city through which the ant *k* passes. Pheromones are a substance released by ants whose concentrations are volatile over time. Visibility, also known as enlightening information, is defined as the reciprocal of the distance between two cities. The calculation formula is as follows:

(2) $$\begin{array}{c} \sigma_{ij}\left(t\right)=\frac{1}{d_{ij}}, \end{array}$$

*σ*_*ij*_(*t*) is the enlightening information between cities *i* and cities *j*, and *d*_*ij*_ is the distance between the two cities. The closer the distance between cities, the greater the enlightening information, and the more likely the path is to be selected. Enlightening information is fixed. *p*_*ij*_^*k*^(*t*) represents the probability of ant *k* moving from city *i* to city *j* at time *t*, and *p*_*ij*_^*k*^(*t*) is defined by the following formula:

(3) $$\begin{array}{c} P_{ij}^{k}\left(t\right)=\left\{\begin{array}{cc} \frac{{\left[\tau_{ij}\left(t\right)\right]}^{\alpha }{\left[\sigma_{ij}\left(t\right)\right]}^{\beta }}{\sum_{j\in J_{k}\left(i\right)}{\left[\tau_{\text{is}}\left(t\right)\right]}^{\alpha }{\left[\sigma_{\text{is}}\left(t\right)\right]}^{\beta }}, & j\in J_{k}\left(i\right),\\ 0, & j\in J_{K}\left(i\right). \end{array}\right. \end{array}$$

In the above formula, *τ*_*ij*_(*t*) is the residual pheromone on the two interurban paths (*i*, *j*) at time *t*, *α* is the information heuristic factor, the importance of the residual information, and *β* is the expected heuristic factor, which is the relative importance of the information. *J*_*k*_(*i*) represents the set of cities to which the next step allows transfer when ant *k* is in city *i*, defined as the following formula:

(4) $$\begin{array}{c} J_{k}\left(i\right)=V-{\text{Tube}}_{K}. \end{array}$$

After each ant foraging visits all cities, the pheromone concentration of the interurban path will change. The pheromone on the path should be updated, and the pheromone update is calculated as below:

(5) $$\begin{array}{c} \tau_{ij}\left(t+1\right)=\left(1-\rho \right)\tau_{ij}\left(t\right)+\Delta \tau_{ij}\left(t\right),\\ \Delta \tau_{ij}\left(t\right)=\sum_{k=1}^{n}\Delta \tau_{ij}^{k}\left(t\right),\\ \Delta \tau_{ij}^{k}\left(t\right)=\left\{\begin{array}{cc} \frac{Q}{L_{k}}, & \text{Ant }K\text{ passes through the path between the two cities}\left(i,\;j\right)\text{,}\\ 0, & \text{If not,} \end{array}\right. \end{array}$$

where Δ*τ*_*ij*_(*t*) is the added pheromone on the path at time *t* (*i*, *j*) and Δ*τ*_*ij*_^*k*^(*t*) is the added pheromone of the ant *k* at time *t* on the path (*i*, *j*). *ρ* is the pheromone evaporation coefficient, and 1 − *ρ* is the persistence coefficient of the pheromone. The amount of pheromone released by the ants is *Q*. The *L*_*k*_ is the total path length that the ant *k* passes through all cities.

### 2.2. Model of Ant Group Algorithm

#### 2.2.1. Abstraction of the Individual Ants

Ant colony algorithm is a computer model of real ant foraging behavior, which is a mechanistic behavior. Therefore, the “ants” in the ant colony algorithm are an abstraction of the real ants, and it is impossible and unnecessary to completely reproduce the individual real ants. Ant abstracted out is known as artificial ant. Artificial ant can be regarded as a simple agent, which can complete the construction process of the simple solution to the problem and can talk with other agents through a means.

Artificial ants and real ants have the following points in common:

1. There is a mechanism in which individuals communicate in a group [14]: pheromone.
2. Complete the same task: to find the shortest path [15].
3. Random selection strategies that use current information for path selection: probabilistic selection strategies.

The abstract artificial ants have their own personality.

1. Artificial ants are present in a discrete space [16].
2. Artificial ants have the memory ability.
3. Artificial ants have the corresponding enlightening information.

It is precisely because of these characteristics that artificial ants make ant colony algorithms have higher intelligence and make them more efficient [17].

#### 2.2.2. Description of the Problem Space

Although real ants in nature exist in a three-dimensional environment, we can completely abstract the ant foraging route on a plane or curved surface [18]. In other words, such a three-dimensional environment can be regarded as a special two-dimensional plane. In addition, if you want to use the ant colony algorithm on the computer, the abstract artificial ant must move in a discrete two-dimensional plane [19, 20]. Real ants forage on continuous paths, so the ant colony algorithm must abstract such continuity into discrete, and this abstraction is understandable. If the actual problem can be described by graphs, you can consider whether the problem can be solved with the ant colony algorithm [21].

#### 2.2.3. Find an Abstraction of the Path

Artificial ants move between nodes in a discrete two-dimensional plane, so through which path does the current node reach the next node? What to choose? In this case, the size of the pheromone on the path can be abstracted into the weights on the edge of the graph [22]. At each node, the artificial ant selects the next node based on the size of the weights on the path. By using this method, reaching the target node from the initial node obtains a feasible solution to the problem.

#### 2.2.4. Abstraction of the Pheromone Volatilization

Ants in nature always leave pheromones continuously along the path, which will gradually evaporate over time. Since the computer handles disengagement events, it can consider the discrete time of the pheromones (i.e., the weight (the pheromone) of the artificial ant) [23].

#### 2.2.5. Introduction of Enlightening Information

The above abstraction is just a computerized representation of the real ant colony foraging behavior, reflecting the very high self-organization of ant colony foraging behavior [24]. However, this self-organization takes a lot of time, often this time-consuming is unacceptable, and we must improve the time utilization. The ant colony algorithm is given a guide according to the specific features of the problem space. Introduce heuristic information within the probabilistic strategy that determines the direction of an ant walk, giving all ants a prior knowledge about the length of the path.

## 3. Design of the Ant Colony Algorithm

To solve the scheduling problem, the ant colony algorithm [25–31] needs to establish a special graphical structure. From the analysis of the scheduling problem, the relationship between the five elements of curriculum, teacher, class, classroom, and time in the scheduling process turns into the relationship between OTL “Courses, Teachers, Classes” and RI “Classroom, Time.” Using the binary graph model can solve the maximum matching problem of two mutually disjoint OTL sets and RI sets. The structural course scheduling problem satisfies the hard constraints of the binary graph model, and can fulfill the basic requirements of the course scheduling problem, that is, the class plan is at the right vertex, the classroom meets the needs of all courses, and does not conflict with the teacher, class, and course time. However, to optimize the course scheduling, we need to use ant colony algorithm for further dynamic adjustment.

### 3.1. Bipartite Model

The bipartite graph [32–35] is that for an undirected graph, the set of vertices can be divided into two disjoint subsets, and the two vertices attached to each edge belong to these two different subsets.

#### 3.1.1. Set of Vertices of the Model

On the basis of analyzing the problem of class scheduling, it is found that before class scheduling, each department should provide <course, class and teacher> relationship to the general academic affairs office, and the academic affairs office of the general school should arrange the class <time and classroom>. Here, we reduce the scheduling problem to be searched in the solution space of *O* × *T* × *L* × *R* × *I* Cartesian product to solve the maximum matching problem formed by <course, class, teacher> relationship and <time, classroom> relationship.

Suppose < *R*_*OTL*_ representative for course, class, teacher> relationship, <time, classroom> *R*_*RI*_

All tuples in the *R*_*OTL*_ relationship are regarded as all vertices on the left of the bipartite graph, actually the constituent vertex set *G*_*OTL*_, and all tuples in the *R*_*RI*_ relationship are regarded as all vertices on the right of the bipartite graph, actually the constituent vertex set *G*_*RI*_. So, any two vertices belonging to set *G*_*OTL*_ are independent between them. Similarly, any two vertices belonging to set *G*_*RI*_ are also independent. In addition, the quantitative relationship between sets |*G*_*OTL*_| and |*G*_*RI*_| must meet |*G*_*OTL*_||*G*_*OTL*_| < |*G*_*RI*_|; otherwise, the scheduling problem must not be solved. |*G*_*OTL*_| represents the number of courses to be scheduled, and |*G*_*RI*_| represents the number of time-space resources that can be provided. Not only that, but |*G*_*OTL*_| ＜ |*G*_*RI*_| should be guaranteed, so that enough <time-space> for students to self-study. Let *N* be a set of all vertices in the bipartite graph model of the scheduling problem with the following formula:

(6) $$\begin{array}{c} N=G_{OTL}\cup G_{RI}. \end{array}$$

#### 3.1.2. The Set of Edges of the Model

First, because the curriculum must be arranged in the desired type of classroom, not all vertices in *G*_*OTL*_ are full mapping to all vertices in *G*_*RI*_, and only classrooms that meet the requirements can be connected to the corresponding curriculum. So, the edges in the bipartite graph are the connections of all nodes in *G*_*OTL*_ and all nodes in which matching the classroom type matches *G*_*RI*_. Secondly, the classroom capacity allocated during the schedule must meet the number of students in the class, so the second part diagram should not include the classroom capacity that does not meet the class size. Thirdly, the course of a class must be arranged in the campus where the class is located, so the edges of the two diagram must meet the same campus attributes of the left and right vertices. Assuming that *S* is a set of edges in the bipartite graph model of the scheduling problem, then *S* = {(*n*_1_,*n*_2_)|*n*_1_ ∈ *G*_*OTL*_ (*n*_2_ ∈ *G*_*RI*_). The type of classrooms required in course n1 matches the type of classrooms provided by *n*_2_, the number of classes taught in course *n*_1_ is <the number of seats provided by *n*_2_, and the campus attributes of the class in course *n*_1_ match the campus where the classroom provided by *n*_2_ is located.

#### 3.1.3. The Weights of Each Side of the Bipartite Graph

The weights on each edge can be constructed according to the expected degree of a specific course on the correlation <time, classroom> in the scheduling question. If in order to ensure the teaching effect, higher mathematics class had better be arranged in 1-2 morning. In this way, the weights (cost *c*_*ij*_) of all the higher math classes in the left set *G*_*OTL*_ in the bipartite graph and all the nodes in the 1-2 am set *G*_*RI*_ are less than the weights of the nodes in the other time periods. In this way, these edges are selected to the better solution.

#### 3.1.4. Bipartite Model

**Figure 1:**
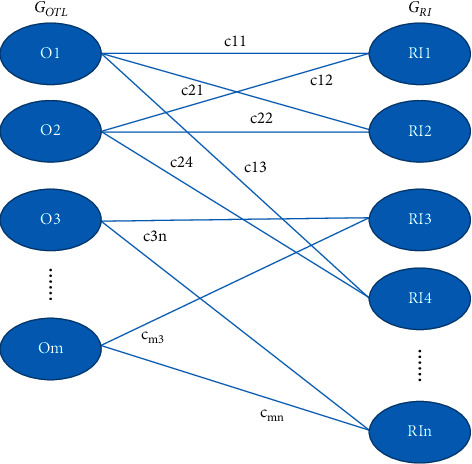
Bipartite graph model of lesson scheduling problems.

The bipartite model for the scheduling problem, *G* = {*N*, *S*, *C*}, is shown in Figure 1.

### 3.2. Determine the Bipartite Graph

**Figure 2:**
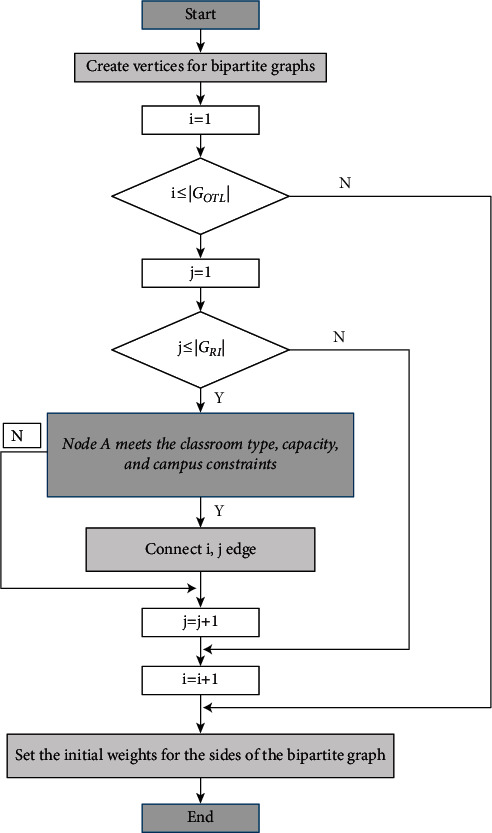
The bipartite graph model of the scheduling problem is constructed into a graph.

The bipartite graph model of the scheduling problem is constructed into a graph as shown in Figure 2.

### 3.3. Ant Group Algorithm Design in Scheduling

#### 3.3.1. Structure of the Individual Ants

Every ant must have a memory ability

1. Each ant can remember which <time, classroom> nodes in *G*_*RI*_ have completed the schedule and which nodes are still in the waiting stage. The specific method is to assign each ant an RI allocation table, which records which <*R*, *I*> nodes the ant passes through during this tour, and updates every node. Also, the <*R*, *I*>allocation table needs to be emptied in time before the ant starts its next trip.
2. In the process of a weekly tour, the distribution of all courses <time and classroom> can be used to compare with other ant results to select the optimal class scheduling method. The implementation was made by assigning a temporary schedule to each ant. For each course, a match is written into a temporary schedule. When you end the tour, compare the cost of all the ants' temporary schedules and find the one with the least cost. Clear out again as all the ants begin a new round of travel.
3. Ability to remember the closeness of course nodes and <time, classroom> nodes. All ants used the same weight information table, used to record this closeness.

Each ant should have a priori ability when choosing a path.

Ants have a full understanding that it is inappropriate to arrange a class in a certain classroom or in a certain period of time. The method is to make the generation value of the edge in the bipartite graph play a full role. Moreover, this generation value directly reflects the expected degree of correlation between the class nodes and <time and classroom> nodes.

Each ant communicates with each other by the pheromone left on the path passing through.

With the process of ant optimization, the amount of information on some paths becomes more informative than others. The amount of information can guide the ants to find the optimal solution.

The ants follow a probabilistic selection strategy to perform path selection.

The probabilistic selection strategy also increases the possibility of random selection of paths and avoids the premature ending of algorithms in bad solutions.

#### 3.3.2. A Weekly Tour by the Ants

Ants complete a search for all the nodes in the graph, called a weekly walk. The global optimal route is the route that results the best of all the searches you have done. In the classic TSP problem, ants start from the initial city node and pass through each city node in the graph and only once. When all the nodes finish, the ant's one tour ends. The definition of ant travel in the scheduling problem is different in the bipartite model of the scheduling problem, and all ants start from a node in the left *G*_*OTL*_ set of the bipartite graph to find the corresponding matching node in the right *G*_*RI*_ set according to the state transition function. Then, all the ants return to the next node in the *G*_*OTL*_ set to start the next search for matches, logically regarded as a node at the beginning of the next one and the previous one. It was not until the ants matched all the courses in set *G*_*OTL*_ that the weekly tour was over (of course, there must be some knots in *G*_*RI*_ at the end of an ant's tour).

#### 3.3.3. The Pheromone Strategy—The MMAS Pheromone Strategy Was Used

1. Only the ants with the least cost perform pheromone updates in each iteration, a practice that enables the algorithm faster convergence to the better solution.
2. The premature stagnation of the algorithm appears because pheromones are too concentrated on several better paths, while other paths are because no ants pass by for a long time, and pheromones are gradually volatile, resulting in the pheromone concentration on the path to 0. Exploring new paths is almost impossible. To reduce the possibility of stagnation in the early algorithm search, pheromones are restricted to range [*τ*_min_, *τ*_max_], which can easily avoid pheromones becoming too large or too small in the course of the algorithm running. Clearly point out *τ*_*ij*_∈[*τ*_min_, *τ*_max_]. In the process of ant optimization, if *τ*_*ij*_ ≥ *τ*_max_, then make *τ*_*ij*_ = *τ*_max_; if over *τ*_*ij*_ ≤ *τ*_min_, the order *τ*_*ij*_ = *τ*_min_. This effectively avoids premature stagnation and expands the search space of the algorithm.
3. Each path initializes the pheromone concentration to *τ*_max_, and doing so can increase the search range of the solution in the beginning of the algorithm.
4. The simultaneous introduction of a pheromone smoothing processing mechanism, namely, Δ*τ* ∝ (*τ*_max_ − *τ*_*ij*_(*t*)), the pheromone enhancement obtained by paths with already high pheromone concentration, will facilitate efficient path search for paths smaller than the pheromone concentration.

#### 3.3.4. Scheduling Steps

1. Refer to the course schedule, divide the five elements of the scheduling problem into two vertex sets OTL and RI, then connect the vertices in the OTL and RI sets that meet the conditions, build the common weight information table of vertices on both sides, and establish a binary graph model.
2. The parameters are information heuristic factor, expected heuristic factor, pheromone evaporation coefficient, cycle iteration number, and pheromone concentration recording table in the initialized ant colony algorithm.
3. Initialize all teacher time allocation table, class time allocation table, and classroom time allocation table; initialize the temporary class table, individual weights, taboo table, visited vertex set, and classroom and time allocation table in RI.
4. Determine whether the iterative optimization is over. If the CNT ≤ MAXITER result is false, jump to step 11.
5. All ants will randomly assign a vertex in the OTL set, initializing the individual weights and schedules for each ant.
6. Determine whether each ant has visited all vertices in the OTL set; if all vertices are visited, jump to step 10; otherwise, randomly select a vertex that is not visited.
7. The transition probability A was calculated for each ant.
8. Determine whether the hard constraint is met. If there is a conflict, jump to step 7 to select the path.
9. Record the selected path, modify the ant individual class table, taboo table, visited vertex set, and individual weights, and jump to step 6.
10. Update the pheromone concentration value of the binary ad model and jump to step 3.
11. Output individual weights with the least cost.

### 3.4. Flowchart of Automatic Class Scheduling

**Figure 3:**
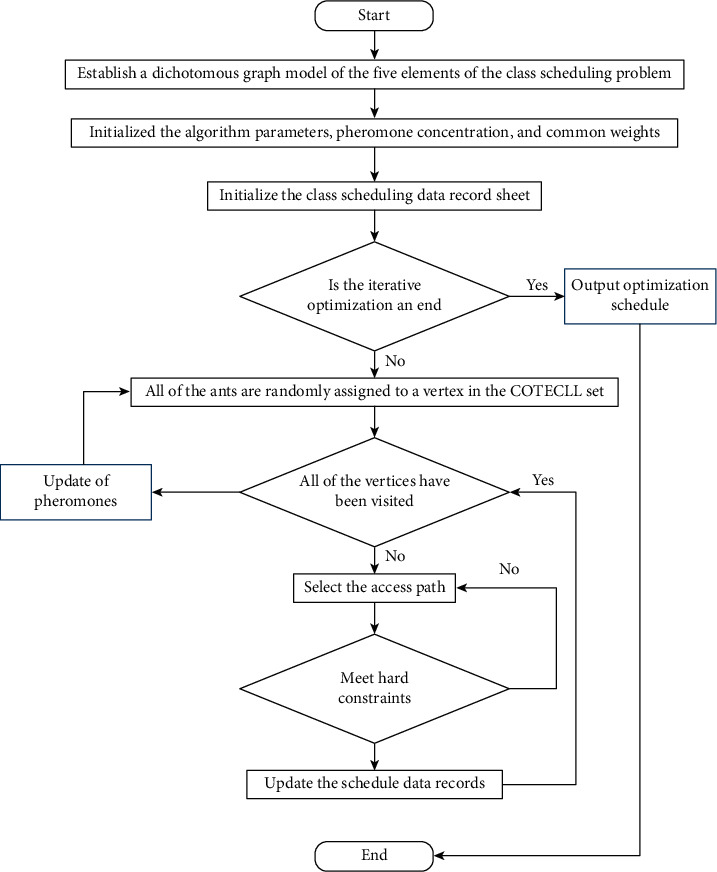
Flowchart of automatic class scheduling of ant colony algorithm.

Flowchart of automatic class scheduling under the ant colony algorithm is shown in Figure 3.

## 4. Conclusion

With the gradual expansion of college enrollment scale and the more and more popularization of computer in teaching, it has become one of the urgent research topics to be solved in teaching management to replace the manual class scheduling with high labor intensity and low work efficiency in teaching management. According to the characteristics of the scheduling problem, the paper abstracts it into a bipartite graph model and applies the ant colony algorithm to match the bipartite graph to find the better arrangement method of the course. The main achievements are as follows:

1. The constraints of the scheduling problem are discussed systematically, and we describe the scheduling problem and its constraints with a mathematical model.
2. The scheduling problem is transformed into a bipartite graph model, and the memory ability, ability to master prior knowledge, adaptation ability, and collaboration ability are applied to move back and forth in the two sets of the bipartite graph to complete the schedule arrangement with higher quality.
3. Adaptive selection strategy combining deterministic selection and random selection, dynamically adjusting the probability of deterministic selection and random selection use, maximum and minimalistic pheromone strategy, and pheromone smoothing processing mechanism are applied to the scheduling ant colony algorithm. Experimental results show that this method guarantees the quality of the efficiency and reconciliation of the algorithm.
4. When solving the constraints of the scheduling problem, the ants are endowed with a lot of memory ability and necessary prior knowledge mastery ability, effectively satisfying most of the individual inspired information strategies introduced at the same time, and well solving the problem of a certain time interval in the course arrangement of more than 2 weeks.

The scheduling problem is a complete NP problem, and this paper establishes a bipartite graph model for the scheduling problem. The proposed model is able to greatly reduce the complexity of the algorithm and reduce the solution time from exponential growth to polynomial growth. The experimental results show that the scheduling results using this paper method are ideal, solving all the hard conflicts mentioned here, and also satisfying some soft constraints to the maximum. However, many practical problems are combined classes, special weeks, elective courses, and time constraints on different campuses. Therefore, the algorithm needs to be further improved. I hope my research can provide a new idea for the solution of class scheduling algorithm and contribute to the application of ant colony algorithm.

## Data Availability

The dataset can be accessed from the corresponding author upon request.
